# A multi-stage neural network approach for coronary 3D reconstruction from uncalibrated X-ray angiography images

**DOI:** 10.1038/s41598-023-44633-2

**Published:** 2023-10-16

**Authors:** Kritika Iyer, Brahmajee K. Nallamothu, C. Alberto Figueroa, Raj R. Nadakuditi

**Affiliations:** University of Michigan, 2800 Plymouth Road Building 20-210W, Ann Arbor, MI 48109 USA

**Keywords:** Acute coronary syndromes, Computer science, Biomedical engineering, Fluid dynamics, Computational science

## Abstract

We present a multi-stage neural network approach for 3D reconstruction of coronary artery trees from uncalibrated 2D X-ray angiography images. This method uses several binarized images from different angles to reconstruct a 3D coronary tree without any knowledge of image acquisition parameters. The method consists of a single backbone network and separate stages for vessel centerline and radius reconstruction. The output is an analytical matrix representation of the coronary tree suitable for downstream applications such as hemodynamic modeling of local vessel narrowing (i.e., stenosis). The network was trained using a dataset of synthetic coronary trees from a vessel generator informed by both clinical image data and literature values on coronary anatomy. Our multi-stage network achieved sub-pixel accuracy in reconstructing vessel radius (RMSE = 0.16 ± 0.07 mm) and stenosis radius (MAE = 0.27 ± 0.18 mm), the most important feature used to inform diagnostic decisions. The network also led to 52% and 38% reduction in vessel centerline reconstruction errors compared to a single-stage network and projective geometry-based methods, respectively. Our method demonstrated robustness to overcome challenges such as vessel foreshortening or overlap in the input images. This work is an important step towards automated analysis of anatomic and functional disease severity in the coronary arteries.

## Introduction

The coronary circulation is divided between the right and left coronary trees, each of which supply blood to different regions of the heart muscle^1^. Coronary Artery Disease (CAD) occurs when atherosclerotic plaque accumulates in the coronary arteries, leading to a local narrowing known as stenosis^2^. The most common imaging modality used to diagnose CAD is X-ray angiography, in which 2D X-ray images are acquired as radio-opaque dye is injected into the coronaries. This allows the cardiologist to visualize and estimate diameter reduction at regions of stenosis.

Although anatomical assessment of stenosis severity is the most common diagnostic practice, functional metrics which take hemodynamic parameters into account through the use of invasive pressure wires placed in the coronaries, such as fractional flow reserve (FFR) and instantaneous wave-free ratio (iFR), have led to better diagnostic outcomes^3,4^. In recent years, there have been numerous efforts to derive computational estimates of those metrics or to propose new metrics such as quantitative flow ratio (QFR) that do not rely on invasive pressure wires^5–10^. A key step in generating computational estimates through modeling approaches is defining the coronary tree geometry of the patient. Creating such a 3D model is relatively straightforward when using 3D medical image data such as computed tomography angiography (CTA). However, 2D X-ray angiography requires a method to accurately reconstruct the 3D geometry from a series of images.

Many groups have proposed coronary reconstruction algorithms using the principles of projective geometry and stereovision^11–25^. An overview of these methods is given in "Related work". Despite their success, projection-based methods have several limitations such as their reliance on user input to identify corresponding vessels or features in all the input images. They also either require precise hardware calibration during image acquisition or algorithms to correct the recorded acquisition angles, since recorded angles and distances can have up to a 10% tolerance^26^. Furthermore, projection-based methods are susceptible to how clearly visible all coronary branches are in each angiography image. For example, overlapping branches and vessel foreshortening are two challenges which can introduce uncertainty into the reconstructed 3D geometry.

Recently, a few groups have performed 3D reconstruction of coronary trees from X-ray angiography images using a combination of machine learning and projective geometry. Bappy et al.^27^ and Jiang et al.^9^ used neural networks to automate coronary segmentation, a key pre-processing step to identify the vessels in the angiography images. They then used projective geometry methods to reconstruct the 3D coronary tree given the segmented angiography images as input. This approach eliminated some drawbacks of previous methods by automating vessel segmentation and point correspondence; however, they are still susceptible to errors in recorded image acquisition angle or reconstruction error due to vessel foreshortening. A fully machine learning-based method has the potential to perform automated reconstruction without the need for imaging calibration. Unlike traditional projection-based methods, a deep learning method has the potential to perform well even on challenging input images with vessel foreshortening or overlap.

The main contribution of this work is a multi-stage neural network for 3D reconstruction from 2D segmented X-ray angiographic images which outputs an analytical representation of a 3D coronary tree. To our knowledge, this work is the first purely machine learning approach for 3D coronary tree reconstruction using X-ray angiography alone. Both Bappy et al.^27^ and Jiang et al.^9^ used machine learning for vessel segmentation; however, both relied on projective geometry for the 3D reconstruction task. Here, we have used a neural network to directly learn the 3D geometry of a coronary tree without relying on projective geometry principles. The major advantage of this method is that it does not require knowledge of precise imaging angles, thereby eliminating the need for calibration or correction algorithms to determine imaging parameters. Another advantage is that the neural network could potentially overcome limitations in the input image such as vessel foreshortening, which can hamper projection-based reconstruction. The neural network was trained using analytical 3D coronary trees from a synthetic vessel generator. The advantage of such an analytical representation of the geometry is that it makes it easy to perform parametric hemodynamic analyses. A proof-of-concept example of such analysis is also included here.

In this work, we demonstrate smaller reconstruction errors compared to a single-stage neural network ("Multi-stage versus single-stage reconstruction performance") and traditional projection-based methods ("Multi-stage centerline reconstruction against a projection-based method"). Proof-of-concept hemodynamic analyses showed that acceptable errors were achieved for the clinical quantities of interest despite modest errors in the 3D geometric reconstruction ("Reconstruction of coronary trees").

### Related work

We now present an overview of 3D coronary reconstruction from single or biplane X-ray angiography images. Typically, two or three 2D X-ray angiography images are acquired from different angles to visualize the coronary arteries. First, the position of the X-ray sources and image planes must be located in 3D space using the parameters recorded during image acquisition. Then, the 3D geometry of the vessels can then be recovered using one of two broad classes of methods: centerline projection-based methods and deformable model-based methods. A comparison of methods from the last 10 years is given in Table 1. A more in-depth review of these coronary reconstruction methods can be found in^28^.

**Table 1:** A summary of 3D coronary reconstruction methods using X-ray angiography images as input. We limit this table to work from the last 10 years.

Paper	Method type	Image pre-processing	Calibration algorithm	Automated?	Speed
Galassi 2018	Centerline projection	Segmentation (Hessian filters + fast marching with manual seed points), centerline extraction (fast marching)	Imaging parameter optimization algorithm using corresponding branch points in all images	Semi-automated	1–2 min
Vukicevic 2018	Centerline projection	Segmentation (Hessian filter), centerline extraction (fast marching based on manual seed points)	Genetic algorithm to calculate corrected image acquisition parameters	Semi-automated	Not provided
Banerjee 2020	Centerline projection	Segmentation (Hessian filter + active contour segmentation), centerline extraction (fast marching)	Rigid transformations of angiography planes using corresponding branch points	Fully-automated (given corresponding centerline segments in each image)	4 min for centerlines, 10–12 for NURBS surfaces
Yang 2014	Deformable models	Branch endpoint selection (manual), centerline extraction	Calibration via energy-based centerline curve alignment	Semi-automated	Not provided
Cong 2015	Deformable models	Branch endpoint selection (manual)	N/A	Semi-automated	Not provided
Bappy 2021	Hybrid machine learning/projection	Segmentation (neural network), centerline extraction (morphological thinning)	Image acquisition: input images 90 degrees apart, biplane angiography	Fully automated	2 s/vessel branch
Jiang 2021	Hybrid machine learning/projection with multiple imaging modalities	X-ray angiography segmentation (gradient-based) and centerline extraction (minimal path algorithm), IVUS segmentation (neural network)	Image calibration using 3 pairs of matched points, catheter path trajectory mapping for IVUS image alignment	Semi-automated	Not provided
This work	Machine learning	Segmentation	N/A	Fully automated	0.20 s

In the first set of algorithms, epipolar geometry or other constraints are used to identify corresponding vessel centerline points or segments in the 2D angiography images. These points or segments are projected into 3D space to recover the shape of the vessels^11–21^. Many of these algorithms employ segmentation or Hessian-based filtering of the X-ray angiography images as a preprocessing step to identify the 2D vessel centerline points more easily. Meanwhile, the second class of methods projects a template 3D vessel onto the angiographic images and uses an energy function such as generalized gradient vector flow to iteratively deform the 3D template until it aligns with the projected images^22–25^. The initial template is often a cylinder representing a single vessel, which is projected onto each of the input images. An energy function is used to deform the 2D projection to match the angiography images, and the forces from the 2D images are projected and applied to the 3D template. This iterative process is repeated for each branch.

More recently, Bappy et al.^27^ developed a method for automatic coronary reconstruction using biplane angiograms acquired 90° apart. First, a multi-resolution U-Net was used for fast coronary segmentation. Vessel centerlines were extracted and automatically paired using branch endpoints and features, then projected into 3D space. Jiang et al.^9^ used a combination of X-ray angiography and IVUS images for their reconstruction method. Their method involved directly extracting vessel centerlines from the angiography images by calculating the first and second derivatives of the image, and using a deep neural network to segment vessel contours from the IVUS images. Centerlines were projected using point-to-point correspondence to generate the 3D centerline, while IVUS images were re-aligned and used to accurately reconstruct the vessel lumen. While these methods used neural networks for segmentation, they still relied on projective geometry for 3D reconstruction. We aim to improve upon previous work by using neural networks for the 3D reconstruction task itself. In addition to eliminating the need for image calibration procedures or algorithms, a deep learning method would improve the automation and speed of coronary reconstruction.

## Methods

### Multi-stage neural network design

**Figure 1:**
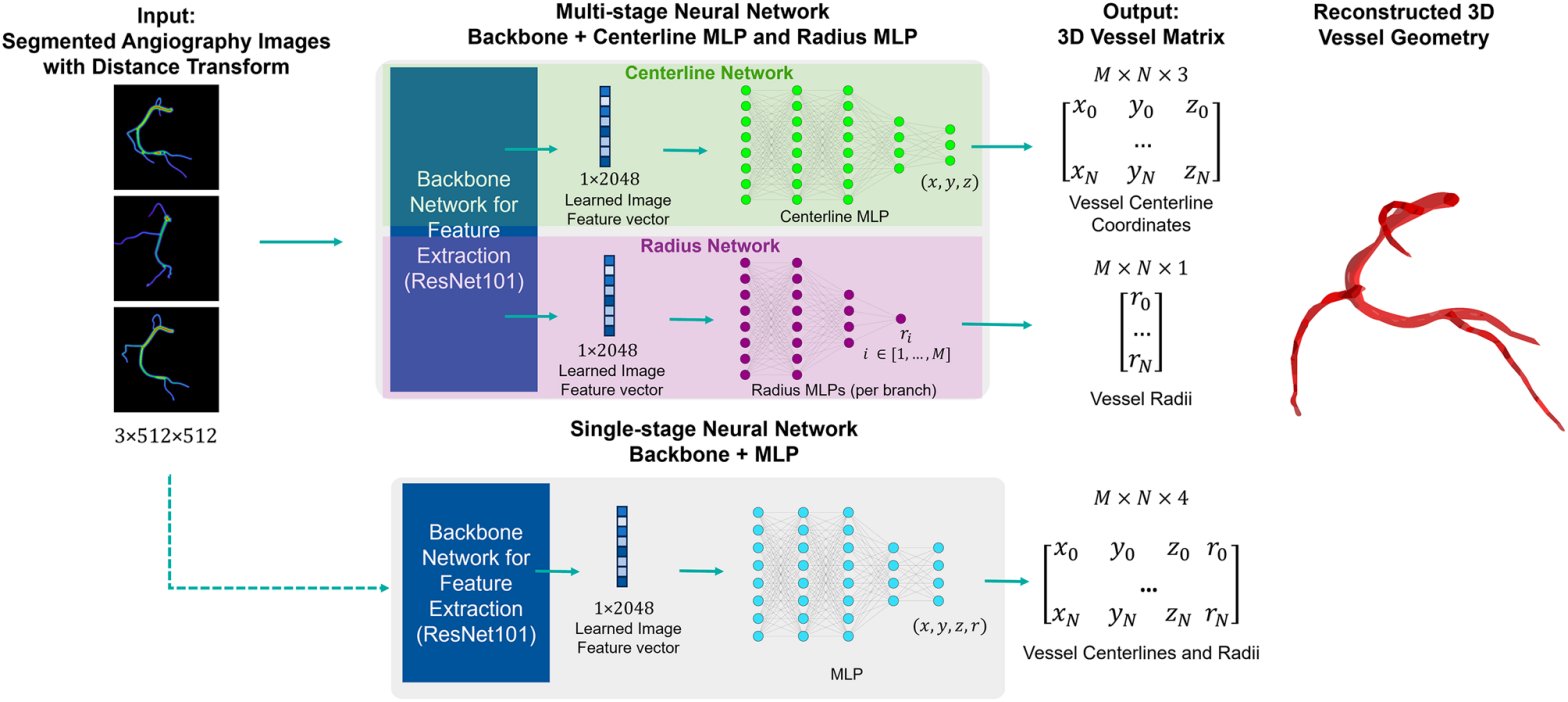
Neural network architecture. The input is a set of segmented X-ray angiography images with a Euclidean distance transform applied for mathematical smoothness. The multi-stage network is composed of a backbone network and separate multi-layer perceptrons which predict vessel centerlines and radii. In contrast, the single-stage network is composed of the backbone network and a single MLP which predicts both vessel centerlines and radii simultaneously. In this paper, the angiography images are created from ground truth synthetic data ("Synthetic dataset generation"), although the network is capable of taking segmented clinical angiograms as input data as well.

We present a multi-stage neural network for 3D reconstruction of coronary trees. In order to reconstruct the 3D coronaries from several 2D angiography images, we must first identify the vessel boundaries or centerlines in each input image. Segmentation algorithms^11,14–17,20^, wave-propagation^12^, spline-fitting to user-defined control points^13,24^, or active contour methods^22,25^ are typically used to delineate the vessels or their centerlines. Therefore, the input to our network is two or three segmented angiograms for a given coronary tree, with a Euclidean distance transform applied to create a smooth field that implicitly encodes both vessel centerlines and radii (see Fig. 1). The input images have dimension 512 × 512, which is the typical size of a clinical X-ray angiogram image. In this work, segmented or binarized angiograms were directly created by our synthetic data generator, where the 0 pixels correspond to the background and the 1 pixels correspond to the vessels ("Synthetic dataset generation"). For clinical angiograms, we have previously developed a vessel segmentation neural network known as AngioNet^29^ which can convert the desired clinical images into segmented binary angiograms. Since no angle or distance information is provided to the neural network as an input, image calibration or parameter correction algorithms are not required.

The multi-stage neural network was designed to reconstruct both vessel centerlines (stage 1) and radii (stage 2) for each branch in the coronary tree. We refer to the network as multi-stage because it has two separate regression heads, or multilayer perceptrons (MLP), to learn the 3D vessel centerline coordinates and radii. This approach is in contrast to a single-stage network with a single regression head to learn both the centerline coordinates and radii. The centerline and radius stages both shared a convolutional neural network backbone to learn relevant features of the coronary tree from the input images. In this work, we chose to use ResNet 101^30^ as the backbone network, however any network that is appropriate for image feature extraction can be substituted. Some examples of other backbone networks are given in "Effect of multi-stage network backbone". While the convolutional layers of the ResNet backbone can learn image-based features relevant to the vessel geometry, a multilayer perceptron (MLP) is better suited to solve the regression problems of identifying the 3D coordinates of the vessel centerline and corresponding radii. Therefore, we replaced the final layer of the backbone network with separate MLPs for the centerline and radius stages, as follows.

In the centerline stage, the final fully connected layer of the backbone network was replaced by a multilayer perceptron (MLP) with Rectified Linear Unit (ReLU) activation and batch normalization between layers. The MLP was composed of 4 hidden layers, where the first 3 layers had 1024 neurons and the last layer had 512 neurons. The output of the centerline MLP was a $$M*N*3$$ linear layer, containing $$N$$ centerline points for each of the $$M$$ branches in the binarized angiogram. This output vector was reshaped into a matrix before computing the loss.

Meanwhile, the radius stage replaced the final layer of the backbone with a separate MLP for each branch, for a total of $M$ MLPs. The radius MLPs were composed of 3 hidden layers with 128 neurons each, with batch normalization and ReLU activation between each hidden layer. The output of each MLP was a vector of radii of dimension $N$. The MLP for each branch was trained separately to improve the network’s ability to capture sudden reductions in vessel radii at regions of stenosis. Without this step, stenoses are likely to be overlooked since they make up a small portion of the points in the coronary tree.

The outputs of both stages were concatenated to form an $M\times N\times 4$ matrix, where the last dimension encodes the 3D centerline coordinate and radius for each point as $(x,y,z,r)$. The centerline points and their corresponding radii in the vessel matrix can be used to compute the surface of each tubular vessel, which is visualized in Fig. 1 as the reconstructed 3D vessel geometry.

The radius stage was trained using the mean squared error as the loss function:

1 $$\begin{array}{c}\frac{1}{n}\sum_{i=0}^{n}{\left({y}_{i}- {\widehat{y}}_{i}\right)}^{2}\end{array}$$

where $$y$$ is the ground truth and $$\widehat{y}$$ is the neural network prediction, and $$n$$ is the total number of points in the coronary trees ($$M*N*batch size$$). Conversely, the centerline stage was trained using the same loss function with an additional length regularization term:

2 $$\begin{array}{c}\frac{1}{n}\sum_{i=0}^{n}{\left({y}_{i}- {\widehat{y}}_{i}\right)}^{2}+ \lambda \sum_{j=0}^{M}{S}_{{y}_{j}}- {S}_{{\widehat{y}}_{j}}\end{array}$$

where $\lambda$ is the regularization rate, ${S}_{y}$ is the overall arclength of each ground truth branch, and ${S}_{\widehat{y}}$ is the arclength of each predicted branch, summed over the total number of branches. This vessel length regularization term was included because vessel length is an important determinant of the pressure gradient through a vessel, an important indicator of disease severity. In this work, $\lambda$ was set to 0.1. In practice, the regularization term should be normalized by the batch size; therefore a $1/batch\_size$ term is also absorbed into $\lambda$.

An Adam optimizer with learning rate 5e−4 and weight decay (L2 regularization) was used to train both stages. Batch size was set to 8 and the multi-stage network was trained for 300 epochs. For the radius MLPs, the backbone was frozen after the initial 300 epochs and each branch MLP was trained for an additional 50 epochs. We did not observe a notable improvement in accuracy when retraining the backbone network for the centerline and radius tasks.

We now present the single-stage counterpart to our multi-stage network for comparison. The single-stage network architecture was composed of the backbone network and a single MLP which outputs an $M\times N\times 4$ matrix containing both the centerlines and their radii (Fig. 1). The loss function of the single-stage network was a weighted MSE loss:

3 $$\begin{array}{c}\frac{1}{n}\sum_{i=0}^{n}\left({\left({y}_{i}- {\widehat{y}}_{i}\right)}^{2}+{\mu ({r}_{i}-\widehat{{r}_{i}})}^{2}\right)\end{array}$$

Here, $y$ and $\widehat{y}$ represent the ground truth and predicted centerline coordinates while $r$ and $\widehat{r}$ represent the radii along the centerlines. The regularization parameter $\mu$ was chosen such that the centerline and radius terms were of the same order of magnitude. The single-stage network was trained using the same hyperparameters as the multi-stage network to make a fair comparison: Adam optimizer with learning rate 5e-4, L2 regularization, and a batch size of 8.

### Synthetic dataset generation

To train the proposed multi-staged neural network, we require hundreds or thousands of ground truth 3D coronary trees and their corresponding segmented 2D angiograms. In practice, this means that we must identify thousands of patients with both 3D CTA data and 2D X-ray angiograms, which is typically not feasible in single-center studies such as ours. Another challenge of using clinical image data as input is that the coronaries deform in each frame of an X-ray angiography series due to the contraction of the heart. This necessitates temporal registration of frames from multiple angiographic series to create a valid set of input images for 3D coronary tree reconstruction. To produce a large enough dataset and eliminate external sources of error such as temporal registration, we devised a method to produce a sufficiently large training dataset consisting of 5000 static 3D coronary tree geometries and their corresponding sets of 2D projections. While we have used synthetic data to train and validate our 3D reconstruction network, the use of synthetic projection images as input does not preclude future clinical application. A segmentation algorithm or neural network such as AngioNet^29^ could be used to convert clinical angiograms obtained during routine patient care in the future into a suitable input for our network.

This work focuses on 3D reconstruction of the right coronary tree as the large anatomical variation in the left coronary arteries^31^ makes reconstruction more challenging. The method includes two steps: (1) a synthetic 3D coronary tree generator, and (2) a projection algorithm to create sets of segmented angiograms. A brief description of these steps is provided next.

**Figure 2:**
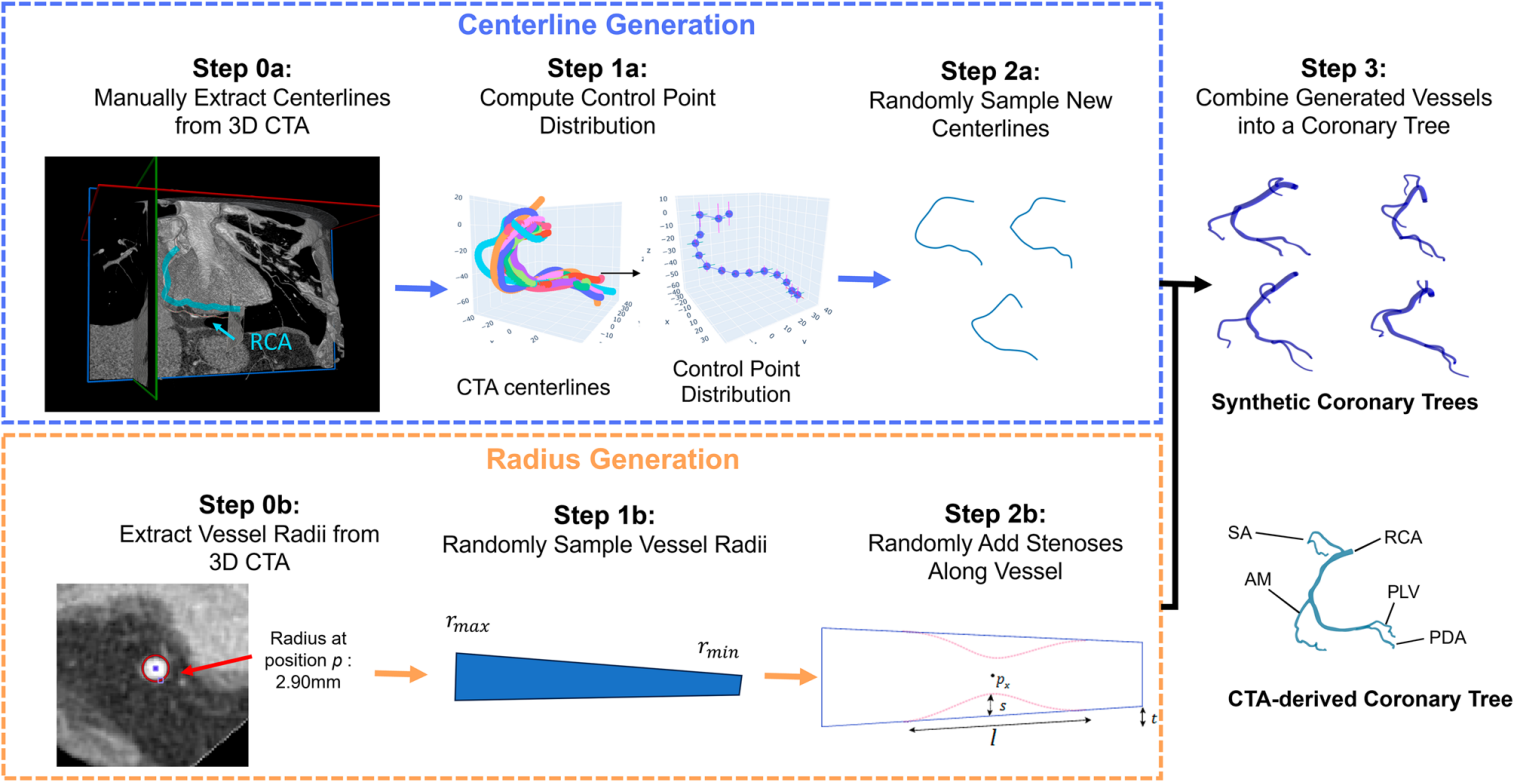
Workflow for synthetic 3D coronary tree generation. In Step 0, we manually identify the centerlines (a) and radii (b) of each vessel in several 3D CTA images. The extracted centerlines are used to determine a distribution of control points (Step 1a) which is used to sample new centerlines for each vessel branch (Step 2a). Meanwhile, the maximum and minimum radius for each vessel are sampled from the range of CTA radius values (Step 1b) and stenoses with a Gaussian profile are randomly introduced (Steb 2b). The vessels defined by the sampled centerlines and radii are combined into a coronary tree in Step 3. Several examples of synthetically generated coronary trees are shown, along with a real patient coronary tree from CTA for comparison. The synthetic trees mimic the structure and shape of the CTA-derive tree while being uniquely different.

Step 1: Synthetic 3D coronary tree generator: Fig. 2 provides an overview of the coronary tree generator. Each synthetic tree was composed of the four main branches of the right coronary tree, namely the right main coronary artery (RCA), sino-atrial node branch (SA), acute marginal branch (AM), and posterior-descending artery (PDA). The posterolateral ventricular branch (PLV) is implicitly included as part of the RCA, which bifurcates into the PDA and PLV branches. The CTA images used to inform the synthetic data generator were acquired at the University of Michigan hospital. All data were de-identified and collected retrospectively according to ethical guidelines, and the study protocol was approved by University of Michigan IRBMED (HUM00155491).

The steps to produce synthetic vessel centerlines and radii were as follows:

1. Vessel centerline splines and radii are manually extracted from 3D CTA images using Cardiovascular Modelling and Simulation (CRIMSON)^32^ software.
2. The range of values for centerline control points and vessel radius (${r}_{max}$, ${r}_{min}$) are determined for each branch from the patient-specific data
3. New centerline points and vessel radii are randomly sampled from the ranges computed in step 1. The centerline spline is interpolated to have $N$ points $(x,y,z)$, creating an $N\times 3$ matrix. The radius vector contains $N$ linearly decreasing radii between ${r}_{max}$ and ${r}_{min}$. Stenoses with 20–90% diameter reduction are randomly introduced in the radius vector.
4. Sampled centerlines and radii for each of the $M$ branches are combined to form a single $M\times N\times 4$ matrix. The centerline points and their corresponding radii can be used to compute and visualize the vessel surface, pictured in Step 3 of Fig. 2.

The resulting coronary trees were augmented with random rotation, shear, and/or warping. This algorithm was refined through repeated iterations with a board-certified interventional cardiologist to generate realistic trees. Further details of the clinical and mathematical assumptions used to inform synthetic data generation are given in Appendix A. Once the coronary tree geometries are defined by the $M\times N\times 4$ matrix, the next step is to create synthetic angiograms of each tree.

**Figure 3:**
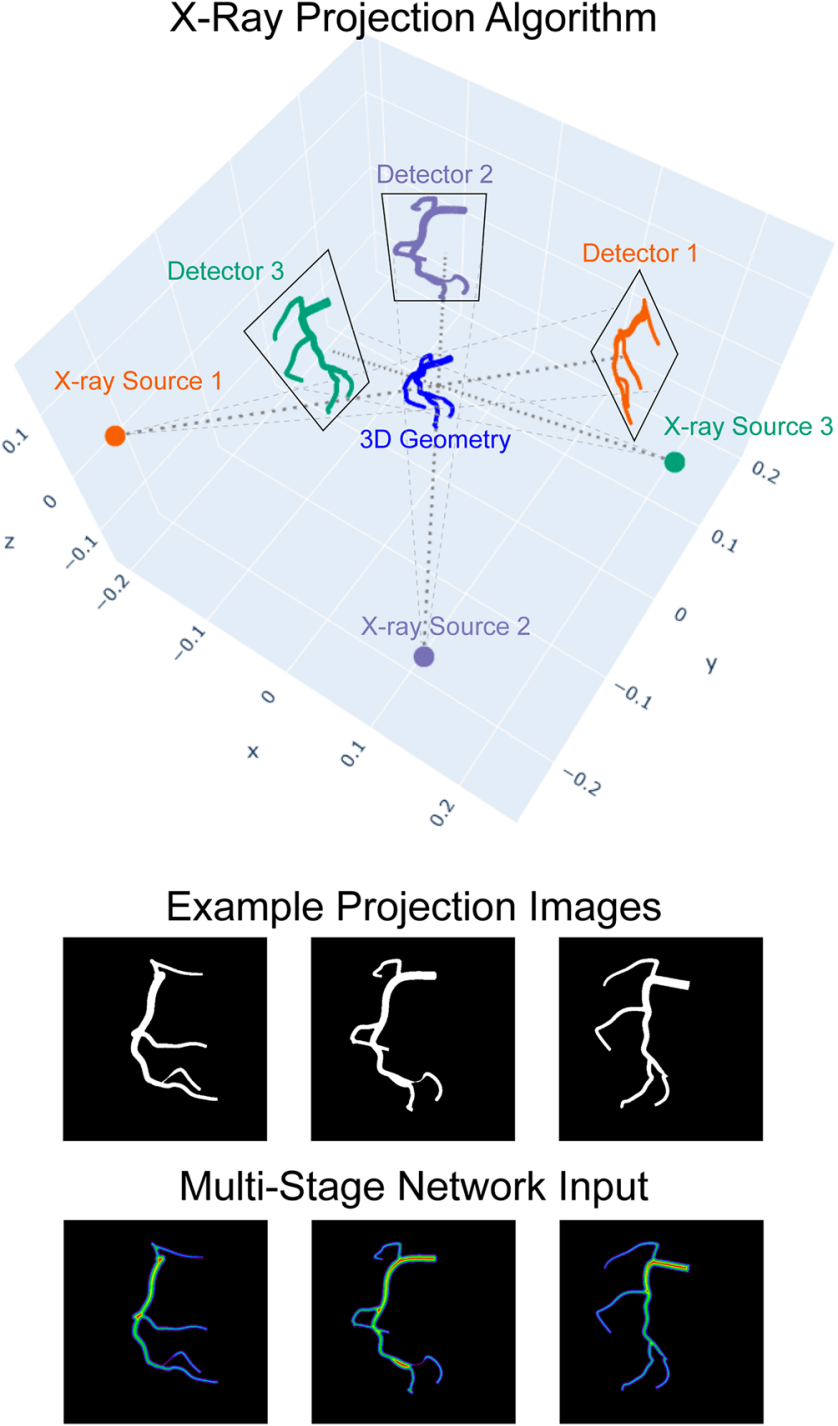
Schematic of synthetic X-ray projection generation. Projections were obtained as a cone beam of X-rays from a point source hitting a detector at a series of sampled random positions. The algorithm produces a set of binary angiograms, each of which is a 512 × 512 image. A Euclidean Distance Transform is applied to the projection images to create the input for the multi-stage neural network.

Step 2: Projection algorithm: Cone-beam projections of each coronary tree were generated from 5 views to mimic the X-ray angiogram acquisition process. In Fig. 3, a 3D coronary tree surface is shown in blue at the origin. The coronary tree surface points are computed from the $M\times N\times 4$ matrix of centerline points and radii. We then determine the placement of the X-ray sources and their corresponding image planes around the 3D coronary tree. Image acquisition angles were randomly sampled from 20-degree windows around commonly used clinical values. A cone beam of rays, marked by gray dotted lines in Fig. 3, are used to project the 3D geometry onto the image planes. Rays that pass through the 3D vessel surface and hit the detector or image plane are marked by a 1 and rays that do not intersect with the 3D model are 0s. This produces the binary 512 × 512 projection images shown in Fig. 3.

Out of the 5 views, 3 were randomly chosen for training. A Euclidean distance transform was applied to the projection images, which were then input into the neural network. The data generated in this fashion were split into 4500 coronary trees and their corresponding projections for training and 500 for validation (90–10 training split). The dataset was thus composed of 5000 total synthetically generated coronary trees, each defined by an $M\times N\times 4$ matrix containing its centerline points and radii (the ground truth), as well as 3 synthetic angiography images from different angles (the network input).

## Results

In this section, we present several methods to evaluate the performance of the proposed method. We first compared the reconstruction error of our multi-stage network against a single-stage neural network to demonstrate the advantages of the multi-stage approach. We then performed a head-to-head comparison between our neural network reconstruction method and a projection-based method. Both comparisons were performed on single vessel geometries for simplicity. Next, we examined the performance of the method in reconstructing right coronary trees. Lastly, an analysis of how geometric reconstruction error affects hemodynamics is presented.

### Multi-stage versus single-stage reconstruction performance

**Figure 4:**
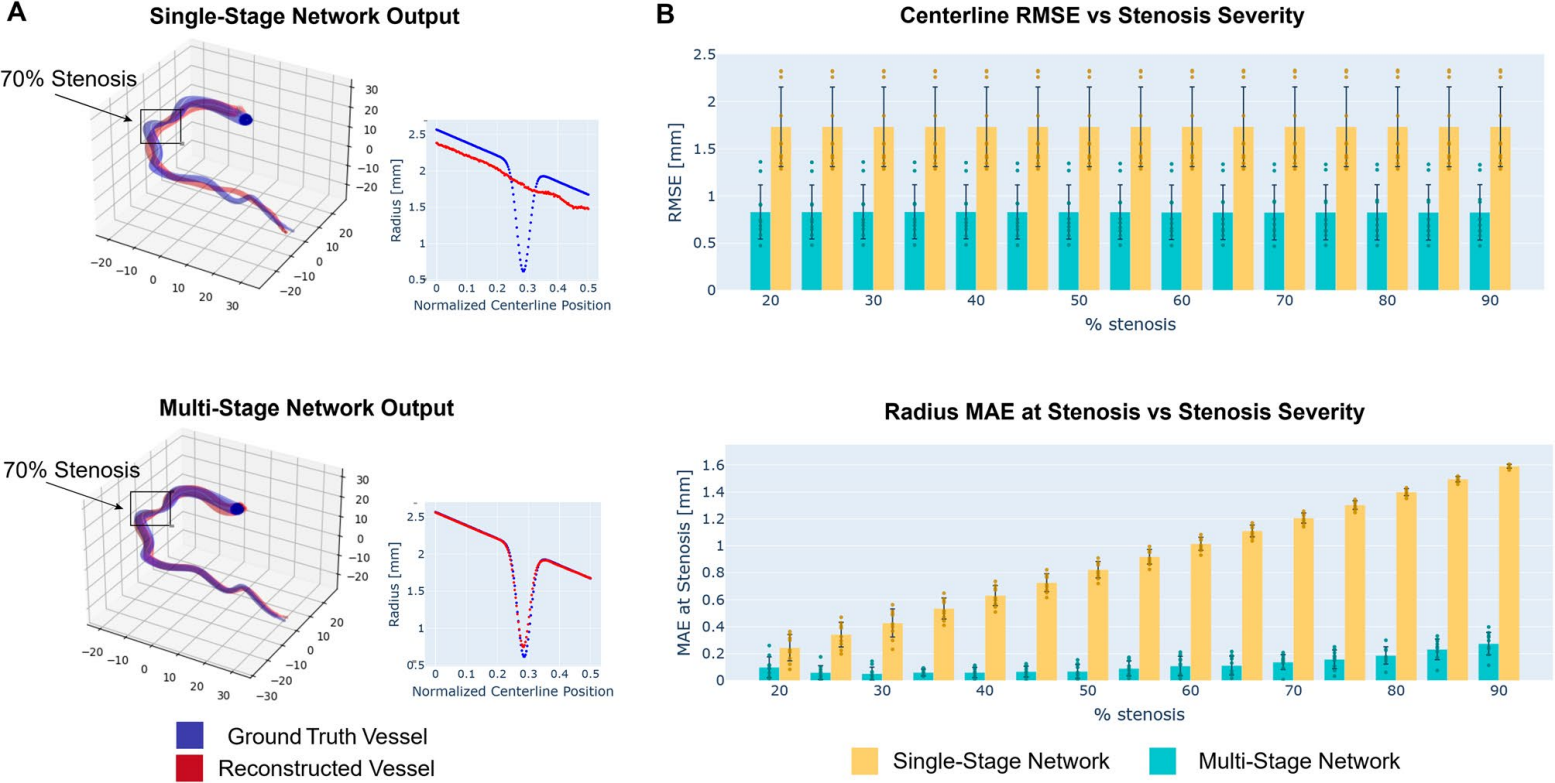
Single- and multi-stage neural network reconstruction results. 2A shows an example vessel with a 70% stenosis and corresponding reconstructions. The single-stage network fails to capture the stenosis while the multi-stage network prediction does capture the stenosis and more closely follows the vessel centerline path. 2B shows reconstruction error for vessel centerlines (RMSE) and radii (stenosis MAE) in 150 synthetically generated vessels with varying levels of stenosis from 20 to 90%. The multi-stage network had a 52% lower centerline error compared to the single-stage network. Furthermore, the single-stage network failed to capture the stenosis, as demonstrated by the increasing radius error for increasing stenosis severity.

A key design feature of our network is its multi-stage nature, which considers the tasks of centerline and radius reconstruction separately. The importance of this feature is illustrated in this section via a comparison between the single- and multi-stage networks. The performance of both networks was evaluated using 10 distinct RCA synthetic vessels. For each synthetic vessel, stenosis between 20 and 90%, with increments of 5%, were introduced, for a total of 150 unique vessel geometries. Reconstruction error was assessed for each geometry. An example of a vessel geometry with 70% stenosis and its single- and multi-stage reconstructions is shown in Fig. 4A. On average, the root mean squared error (RMSE) of the multi-stage network centerline was 52% lower than the single-stage centerline RMSE, see Table 2 and Fig. 4B.

**Table 2:** Centerline and radius reconstruction errors for single- vs multi-stage networks on 10 unique RCA vessel geometries.

	Single-stage	Multi-stage
Centerline MSE [mm]	1.73 ± 0.42	0.83 ± 0.29
Radius MAE at stenosis [mm]	0.927 ± 0.436	0.117 ± 0.068

As for radius reconstruction accuracy, mean absolute error (MAE) at the stenosis was evaluated instead of RMSE in the whole vessel due to the importance of accurately predicting stenosis severity (Table 2). For both networks, the MAE increased with the stenosis severity (Fig. 4B). However, the single-stage network effectively failed to detect the stenosis for all levels of stenosis (Fig. 4A), resulting in extremely large MAE errors (over 1.5 mm) for severe stenoses with over 80% diameter reduction.

### Multi-stage centerline reconstruction against a projection-based method

We now compare the performance of our multi-stage neural network against a projection-based method in the simplest case of a single vessel centerline reconstruction from 2 projection images. In this example, the projection-based approach was a combination of the algorithms proposed by Banerjee et al.^11^ and Vukicevic et al.^12^. Briefly, a point cloud approach^11^ was used to automatically identify up to 10 possible corresponding points for each centerline point in the reference projection image. The set of possible corresponding points was further refined using the reprojection error and ordering constraint cost function proposed in^12^. Matched points from both images were then back-projected and interpolated with a b-spline to create a 3D centerline.

**Figure 5:**
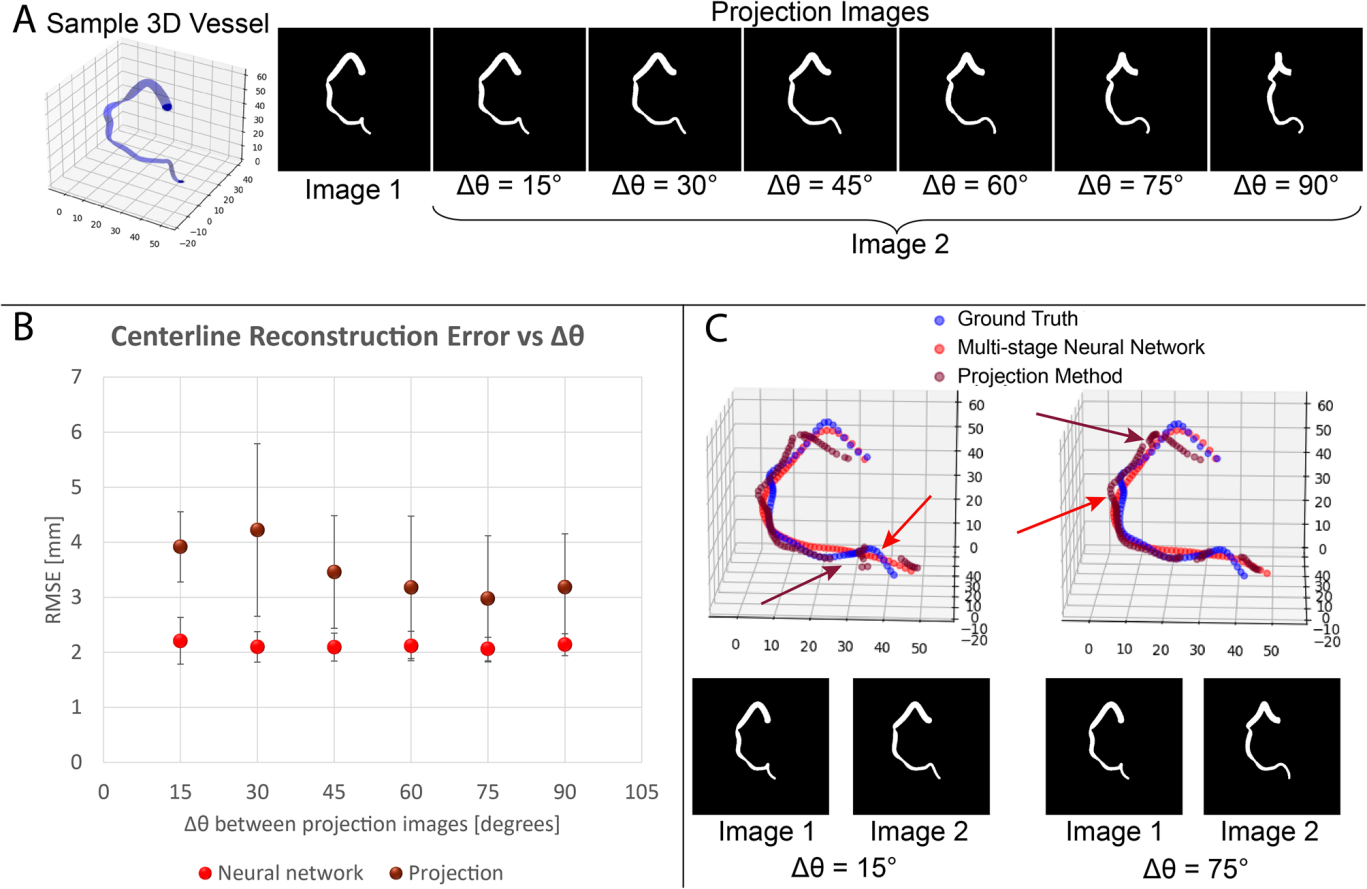
Comparison of neural network and projection-based reconstructions of a single vessel from different input projection images. (**A**) Sample 3D RCA geometry and corresponding projection images. (**B**) The neural network has on average 38% lower reconstruction error for all values of $\mathrm{\Delta \theta }$, the angle between input projection images. (**C**) Examples of the same 3D geometry reconstructed from different input projection images. Red arrows indicate regions where the neural network centerline does not follow the ground truth path while brown arrows indicate regions of uncertainty and gaps in the projection-based centerline.

The dataset of synthetic projection images for this comparison was defined as follows. 5 synthetic RCA vessels were first created. We considered a spherical coordinate system $(\theta ,\phi )$, where $\theta$ and $\phi$ are the azimuthal and elevation angles, respectively. For a given vessel, the first projection image in every pair was fixed at $\theta =-45^\circ , \phi =0$. The second image was defined using six different intervals $\Delta \theta \in [15^\circ , 90^\circ ]$, for a total of 30 different pairs of projection images (5 different vessels, 6 different angles between projection images for each vessel), see Fig. 5A. The reconstruction error as a function of the angle between projection images, $\Delta \theta$, was compared between both methods.

As seen in Fig. 5B, the centerline RMSE and standard deviation was lower in the neural network approach compared to the projection-based method for all tested angles and vessels: 2.12 ± 0.05 mm and 3.49 ± 0.44 mm, respectively. Figure 5C shows two examples of the same vessel reconstructed using 2 pairs of projection images with different $\Delta \theta$. The ground truth centerline (blue), neural network centerline (red), and projection-based centerline (brown) are shown. The neural network centerline followed the average path of the ground truth centerline, although it failed to capture some of the bends along its path (red arrows). Meanwhile, the brown arrows indicate gaps or regions of uncertainty in the centerline reconstructed using projection-based methods.

### Reconstruction of coronary trees

**Figure 6:**
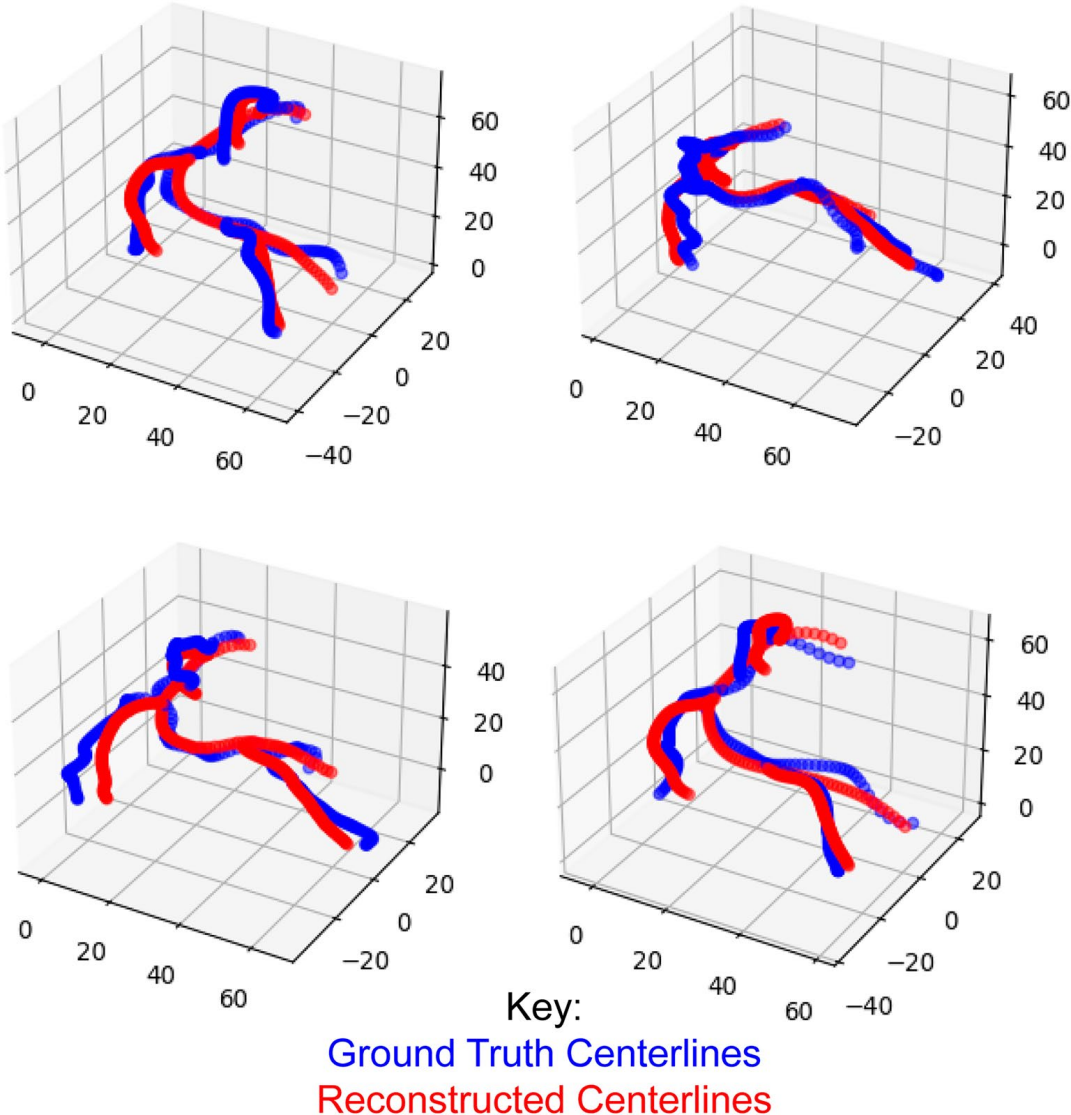
Examples ground truth centerlines (blue), reconstructed centerlines (red), and their corresponding input images. As in the single vessel case, the neural network predictions follow the average path of all vessels but do not have the same tortuosity as the ground truth centerlines.

In this section, we measure the accuracy of the proposed method for coronary tree reconstruction, using the validation set of 500 synthetic coronary trees defined in "Synthetic dataset generation". Figure 6 shows several examples of reconstructed coronary tree centerlines (red) and their corresponding ground truth (blue). We observed that the neural network learned the mean centerline path of each vessel in the tree; however, as in the single vessel case, the predicted vessels were not as tortuous as the ground truth centerlines. The RMSE between the ground truth and predicted centerline points in the validation set was 2.57 ± 0.78 mm. The MAE in vessel length was 8.83 ± 4.81 mm. Optimal values for vessel length reconstruction were obtained with a regularization length parameter $\lambda =0.1$ (see Eq. (2)), which led to a 47% decrease in vessel length error (16.36 ± 2.88 mm) compared to the same network trained without length regularization in the loss function. Larger values of $\lambda$ resulted in over-constraining the vessel length and inaccurate paths for the different branches, whereas smaller values of $\lambda$ resulted in weaker enforcement of the vessel length.

**Figure 7:**
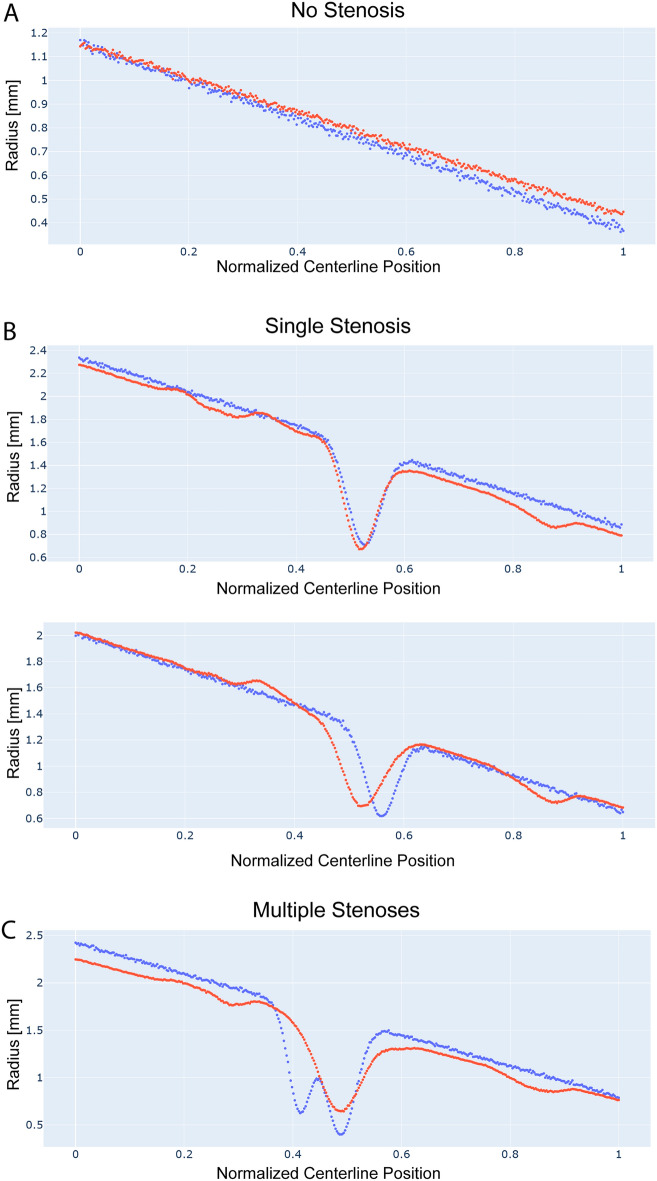
Examples of radius reconstruction (red) along normalized centerline position of the main RCA (blue) in a vessel without stenosis, vessels with a single stenosis, and two stenoses. For the vessel without stenosis, the neural network captures the linear tapering of the vessel well. For the vessels with a single stenosis, the neural network accurately predicts the severity and location of the stenosis, although with some oscillations apparent in regions outside the stenosis, or a slight shift in the stenosis location. Finally, in the multi-stenosis case, the neural network approximates serial stenoses as a single stenosis with an error of 0.3 mm in stenosis severity.

We now consider the error in vessel radius along the centerline for all 2000 branches (500 trees with 4 branches each). The RMSE of the vessel radius was 0.16 ± 0.07 mm, which corresponds to sub-pixel resolution. The error in radius was larger when comparing minimum stenosis diameter (MAE = 0.27 ± 0.18 mm), particularly for severe stenoses greater than 70% diameter reduction, consistent with the behavior reported for single vessel reconstruction in "Multi-stage versus single-stage reconstruction performance". Figure 7 shows examples of radius reconstruction (red dots) along normalized centerline position of the main RCA (blue) in vessels without stenosis (top panel), two cases of a single stenosis (mid panel), and a case with two stenoses (bottom panel). Panel A demonstrates that the neural network accurately captures the tapering of the vessel along its length. Panel B shows that the neural network accurately predicts the severity and location of the stenosis, although with some oscillations apparent in regions outside the stenosis, or a slight shift in the stenosis location. Lastly, in panel C the neural network approximates serial stenoses as a single, longer stenosis with an error of 0.3 mm in stenosis severity. Some oscillations remain apparent in the region outside the stenosis.

### Effect of multi-stage network backbone

In this work, we chose to use ResNet 101 as the feature extraction backbone network; however, other feature extraction networks can also be used in our multi-stage network framework. Here, we investigate the performance of using EfficientNet v2^33^, a smaller backbone network than ResNet 101, and ViT (Vision Transformer)^34^, a larger network and one that uses attention modules instead of convolutional layers for image feature extraction. Table 3 summarizes the centerline MSE and radius MAE at the stenosis for each backbone.

**Table 3:** Centerline and radius reconstruction errors for three different backbone networks: ResNet 101, EfficientNet v2, and ViT.

	ResNet 101	EfficientNet v2	ViT
Centerline MSE [mm]	2.57 ± 0.78	3.00 ± 0.89	2.33 ± 0.59
Radius MSE [mm]	0.16 ± 0.07	0.17 ± 0.06	0.18 ± 0.05
Radius MAE at stenosis [mm]	0.27 ± 0.18	0.78 ± 0.63	0.85 ± 0.44

**Figure 8:**
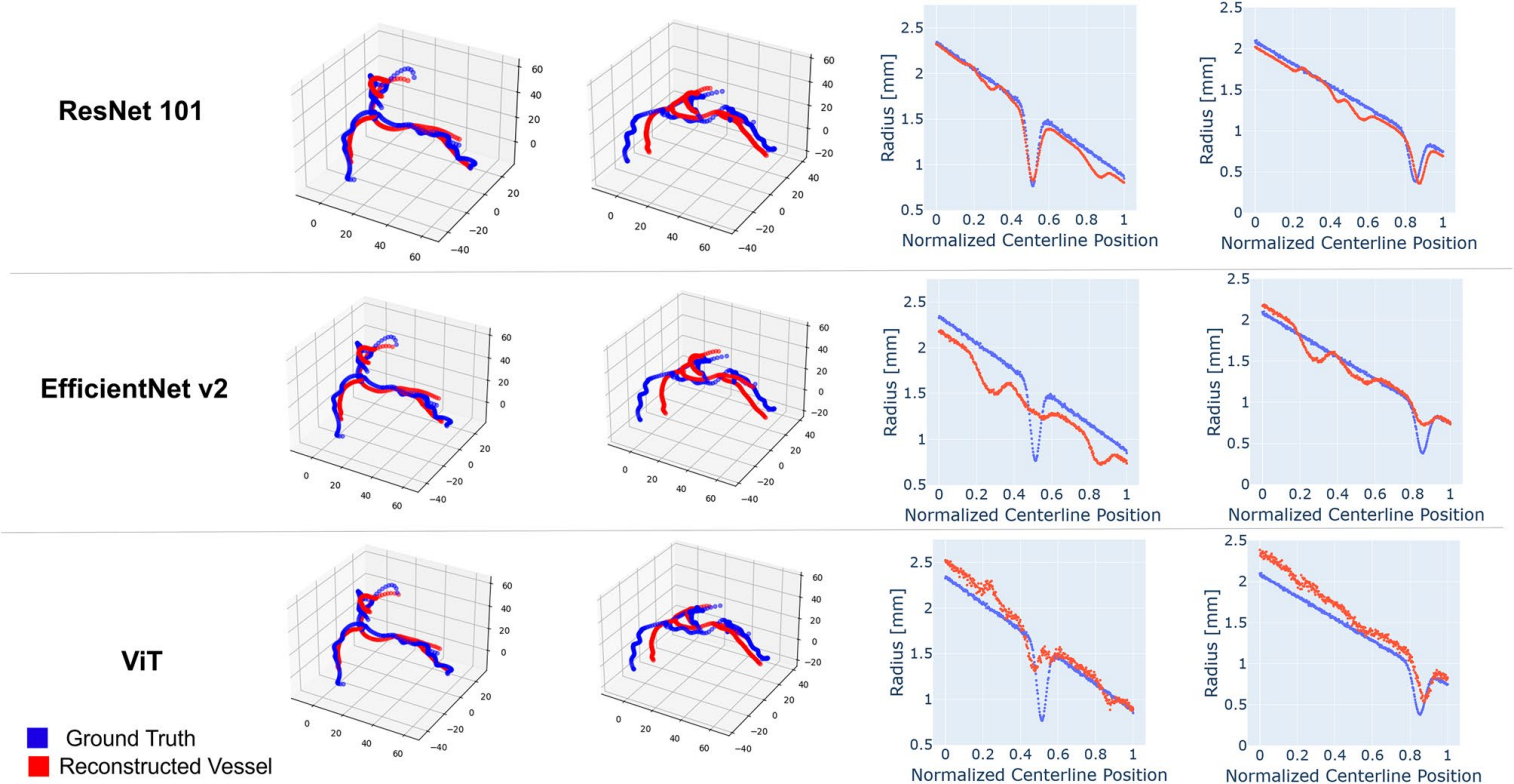
Representative examples of reconstructed coronary tree centerlines and radii using three different feature extraction backbone networks: ResNet 101, EfficientNet v2, and ViT. The ground truth is shown in blue while network predictions are shown in red. All three networks reconstruct similar centerlines that follow the average path of each vessel in the coronary tree. When considering radius along the main RCA branch, ResNet 101 is best able to capture the radius at the stenosis. Meanwhile EfficentNet v2 predicts the same average radius waveform for all vessels, and ViT accurately estimates the location but not the severity of stenoses.

The three networks produce similar errors on centerline reconstruction and overall radius reconstruction, however ResNet 101 achieves the smallest error on stenosis radius reconstruction. Figure 8 shows several representative examples of coronary centerline and radius reconstruction. All three backbones follow the general path of the vessel centerline but fail to capture the tortuosity of the vessels, similar to the results of "Reconstruction of coronary trees". Furthermore, the examples of radius reconstruction show that Efficientnet v2 has not learned the best image features to reconstruct radius since it predicts the same average waveform for all input images. On the other hand, ViT can accurately determine the position but not the severity of stenoses. ViT also demonstrates a systematic error in estimating the maximum vessel radius. ResNet101 outperforms both EfficientNet v2 and ViT at estimating stenosis severity.

### Effect of reconstruction error on hemodynamics

In the previous examples, we established the performance of the multi-stage neural network to reconstruct centerline and radius of vessels in coronary artery trees. However, as stated in the introduction, functional assessment of vessel disease has recently received significant attention. In this section, we investigate the quality of our proposed multi-stage neural network from a functional standpoint. Towards that end, we simulate the physics of blood flow and pressure in the reconstructed coronary trees using computational fluid dynamics (CFD) simulations and compare the results against known ground truth CFD data. Specifically, we compare distributions of pressure down the RCA vessel in ground truth coronary trees (known synthetic geometries) and their reconstructions. The pressure field is the basis to calculate well-established functional metrics of CAD such as FFR, iFR, and QFR^4,6,35^.

**Figure 9:**
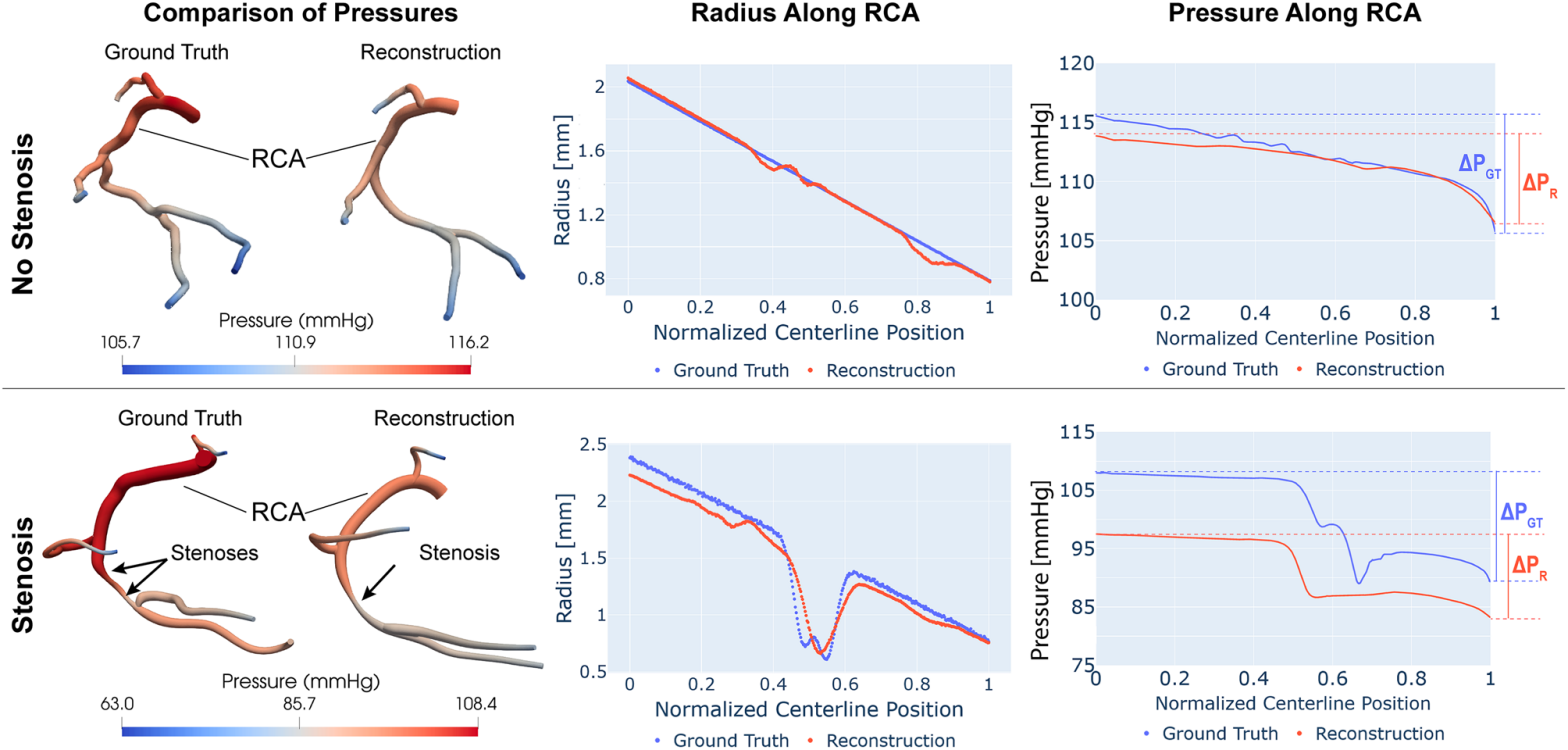
Comparison of pressures in ground truth and reconstructed coronary trees. The top and bottom panels contains results for a healthy tree without stenosis and a disease tree with two stenoses, respectively. The left column shows solution maps of pressure calculated via CFD in ground truth and reconstructed geometries. The center and right columns show plots of reconstructed radius and CFD-derived pressure down the RCA for the ground truth (blue) and reconstructed (red) cases.

CFD simulations with consistent inflow and outflow boundary conditions were run using the validated open-source software CRIMSON^32^. Two examples are discussed: (1) a healthy coronary tree without stenosis, and (2) a diseased tree with one stenosis. Details of simulation parameters (e.g., inflow and outflow boundary conditions, fluid properties such as density and viscosity, etc.) are given in Appendix B. Results are summarized in Fig. 9.

Results for the healthy coronary tree are given in the top panel of Fig. 9. The left column depicts solution maps of pressure calculated via CFD analysis for ground truth and reconstructed geometries. The center and right columns show the radius and pressure, respectively, versus normalized centerline position in the ground truth (blue) and reconstructed (red) geometries. In the top panel, there is no stenosis and the changes in pressure down the RCA are small. The pressure gradients over the ground truth and reconstructed RCA vessels are *DP*_*GT*_ = 9.8 mmHg and *DP*_*R*_ = 8.4 mmHg, resulting in a difference between pressure gradient estimates of $\Delta P=\Delta {P}_{GT}-\Delta {P}_{R}$ = 1.4 mmHg (or only 1.2% of the ground truth inflow pressure).

In the diseased coronary tree, the pressure gradients over the ground truth and reconstructed RCA vessels are DP_GT_ = 18.8 mmHg and DP_R_ = 14.3 mmHg, resulting in an error of $\Delta P$= 4.5 mmHg. Additionally, the pressures in the reconstructed geometry were approximately 10 mmHg lower than the pressures in the ground truth. This is due to the differences in reconstructed radius in the proximal portion of the vessel, as well as the decreased tortuosity (and therefore decreased resistance) of the reconstructed coronary tree compared to the ground truth.

The larger pressure error in the stenosis case compared to the healthy case can be attributed to the radius reconstruction error at the stenosis. Given a vessel with radius $a$, its resistance $R$ is inversely proportional to its radius to the power of four ($R \sim 1/a^{4}$). Thus, relatively small errors in radius reconstruction have substantial impacts on vessel resistance and therefore pressure drop across the vessel. The ground truth stenotic geometry had serial 51% and 57% stenoses. However, the neural network approximated these 2 stenoses as a single, longer, 53% stenosis, corresponding to a radius error of 0.07 mm (see center column Fig. 8). The ratio of pressures on either side of the stenosis (a surrogate of FFR), was 0.886 in the ground truth and 0.895 in the reconstructed vessel. Therefore, despite the 10 mmHg difference in inlet pressure, there was only a 1% error in the clinical quantity of interest.

## Discussion

Reconstructing 3D geometries from sets of 2D image data is a challenging task that often necessitates accurate knowledge of imaging parameters such as angles and distances during image acquisition. Uncertainty of these parameters poses a large challenge for projective geometry approaches to reconstruct the coronaries from X-ray angiography images; therefore, we have developed and tested a novel multi-stage neural network method for 3D coronary reconstruction.

### Considerations of neural network design

The most salient feature of the proposed network is its multi-stage nature, which enables accurate reconstruction of centerline paths and vessel radii. When considering the single-stage approach to reconstructing vessel geometry, we observed that the network was able to accurately capture the trend of linearly decreasing radius along the vessel but could not capture the stenoses ("Multi-stage versus single-stage reconstruction performance"). This is likely because the stenosis radii make up less than 2% of the $N\times 4$ vessel matrix; the stenoses had such a small contribution to the loss function that the network did not learn to predict them. In the multi-stage approach, the stenosis radii had a much larger contribution to the loss function since the mean squared error of the radii was optimized separately from the centerlines. The network was therefore able to learn and accurately reconstruct stenosis severity.

This observation motivated going one step further for the coronary trees and using a separate MLP to learn the radii for each vessel. This was necessary since not all vessel branches contained stenoses; if the network was trained to predict the radii of all branches at the same time, the stenoses would once again make up less than 2% of the $M\times N$ output matrix. A major challenge of reconstructing stenoses is that, while they are the most important diagnostic feature of the image, they only make up a small proportion of the vessel matrix. The multi-stage approach described in this work amplifies the impact of the stenoses and thus improves prediction accuracy.

The size of the MLPs for the centerline and radius tasks was determined by testing networks with a varying number of hidden layers and neurons per layer. Networks with 128, 256, 512, 1024, 2048, and 4096 neurons in each of 1–4 hidden layers were evaluated for both centerline and radius accuracy. For the centerline MLPs, the minimum error was achieved when using 4 hidden layers. The number of neurons per layer did not greatly affect mean squared error, which ranged from 2.72 to 2.91 mm in the validation set of 500 synthetic coronary trees. Therefore, a network with 1024 neurons per layer was chosen as it was of the same order of magnitude as the network output size ($M$*$N$*3).

For the radius MLPs, the lowest error was achieved in networks with 3 hidden layers. Interestingly, although overall mean squared error for the radii was similar for networks with different widths (0.02–0.03 mm), smaller networks performed better at regions of stenosis. The MAE at the stenosis for a network with 128 neurons was 0.27 mm, compared to 0.40 mm and 0.42 mm for networks with 2048 and 4096 neurons, respectively. Thus, we determined that the optimal size for the radius network was 3 hidden layers with 128 neurons each.

### Comparison with projection-based methods of reconstruction

3D reconstruction of coronary trees from 2D X-ray angiographic images has been generally performed using projective geometry and stereovision techniques^11–14,25^. Despite their success, projection-based methods have limitations such as the need to identify corresponding features in the input images, and precise information on the image acquisition angles. Overlapping branches and foreshortening are two key challenges leading to uncertainty in the reconstructed geometry using projective geometry methods.

The key contribution of this paper is a neural network for reconstructing 3D coronary trees that aims to overcome the limitations of projective geometry methods. Our network was trained over a wide range of angles between images $\Delta \theta$ with the purpose of making it robust (and therefore relatively insensitive to this parameter). In "Multi-stage centerline reconstruction against a projection-based method", we compared the performance of our multi-stage neural network against a projection-based method in a synthetic dataset of pairs of projection images with angles between images $\Delta \theta \in \left[15^\circ , 90^\circ \right]$. Our network’s RMSE was on average 39% lower than the projection method across all values of $\Delta \theta ,$ even though the neural network was not trained on images at these specific projection angles. Together with the lower standard deviation, this could indicate that the neural network is more robust to different characteristics of the input images. For instance, different amounts of foreshortening or overlap may occur for a given vessel when projections are taken at different angles. While the projection method reconstructions are less accurate when the input images have high levels of foreshortening or overlap, the neural network could learn universal characteristics of the RCA vessel shape to overcome these challenges and maintain similar accuracy for all input images.

The higher robustness to foreshortening and overlap in the input images of the neural network method is further supported by the qualitative examples in Fig. 5C. The brown arrows, which point to gaps or regions of uncertainty in the projection method centerline, correspond to regions on the input image where the vessel is highly foreshortened or bending at an angle that obscures other parts of the vessel. The neural network interpolates the 3D centerline in these regions, resulting in a continuous path.

The main limitation of the neural network compared to projection methods is that it does not capture all the bends in the vessel centerline path despite accurately following the average path. The lack of tortuosity leads to a shorter reconstructed vessel than the ground truth, despite the length regularization term in the loss function (Eq. 2). This behavior could be a consequence of the network learning the features of a typical 3D RCA shape. While these features ensure that the network can interpolate the 3D centerline even when the input images are challenging, they also may cause the network to predict similar, smooth centerline paths for all inputs. Meanwhile, the projection-based method can accurately capture the details specific to each centerline path using the features of the input images.

The neural network was trained using a MSE loss, which only accounts for point-wise differences between ground truth and reconstructed centerlines. A loss function that incorporates more global shape information such as curvature in addition to the vessel length regularization could improve the centerline path accuracy.

### Reconstruction of coronary trees

When applying the multi-stage neural network to reconstructing a whole coronary tree, the reconstructions captured the average path of all branches in the tree but under-estimated their tortuosity, similar to the behavior observed for single-vessel reconstruction. This appears to correspond to a well-known curve-fitting regression phenomenon: when the network is not large enough to capture the full complexity of a dataset, it tends to predict its average behavior. We hypothesized that increasing the complexity of the neural network by increasing the size of the MLP may alleviate this issue. We tested this hypothesis by performing a scaling study in "Considerations of neural network design" to test the performance of networks with 128–4096 neurons in each of 1–4 hidden layers. From this scaling study, we observed 1% decrease in RMSE between the 4096 × 4 network and 128 × 1 network. Therefore, the computational overhead of increasing the MLP size did not justify the modest decrease in error. As discussed in "Comparison with projection-based methods of reconstruction", a loss function which incorporates global shape may be a more effective strategy to improve centerline accuracy.

As seen in "Effect of multi-stage network backbone", changing the backbone of the multi-stage neural network does little to affect the quality of centerline reconstruction. Since the tested backbone networks were of different sizes and classes (convolutional networks: ResNet and EfficientNet v2, transformer: ViT), this further indicates that changing the loss function or the encoding of the centerline coordinates may be more effective than simply changing the network architecture.

Another challenge of coronary tree reconstruction compared to single vessel reconstruction was to accurately predict stenoses radius. As described in "Considerations of neural network design", employing a multi-stage approach, and training a separate MLP for each vessel branch enabled the network to learn how to predict stenoses. Despite this, the MAE at the stenosis was higher for coronary trees (0.27 mm) compared to a single vessel (0.12 mm). This may be due to the random projection angles, as overlapping branches may have obscured the stenosis in some of the input images.

While changing the network backbone had little effect on centerline coordinate reconstruction, a much larger effect was observed in stenosis radius reconstruction. In Fig. 8, we observe that EfficientNet v2 is unable to learn the nuances of vessel radius from the image features, and thus predicts the same output for all inputs. Since it was the smallest network tested, we can conclude that the backbone network must be sufficiently large to distinguish details such as stenosis radius from the image features. Although ViT was a larger network than ResNet 101, it also failed to accurately capture stenosis radius. The reason for this may be that the input for a transformer network is down-sampled, in this case from a 512 × 512 image to a 385 × 385 image, to reduce the computational complexity of the attention mechanism. The down-sampling step may obscure the relevant radius information implicit in the input images. Transformers could still be effective feature extraction networks, perhaps coupled with convolutional layers as a pre-processing step to reduce the dimensions of the input without losing information.

### Effect of reconstruction error on hemodynamics

The last application example explored the impact of geometric reconstruction errors on hemodynamic indices such as pressure. The simple test examples demonstrated that gradients of pressure (a surrogate of FFR) compared well between ground truth and reconstructed geometries, even though the neural network centerlines were not as tortuous as the ground truth.

The error in radius had a much larger impact on the pressure drop than the centerline error, particularly errors in stenosis radius. As seen in the stenosis example, the neural network approximated two serial stenoses (51% and 57% radius reduction) as a single stenosis with 53% radius reduction. Despite only a 0.07 mm difference in stenosis radius, there was a noticeable difference in pressure profiles across the stenosis between the 2 geometries. However, the gradients of pressure across the stenosis were relatively similar between the 2 cases (less than 1% difference). We hypothesize that accurately capturing the overall vessel lengths and stenosis radii contributed to the accurate estimation of gradients across the vessels. However, at higher levels of stenosis, even small errors in reconstructed radius can lead to large differences in pressure gradients across the vessels.

Given the high sensitivity of vessel resistance to the reconstructed vessel radius ($R\sim 1/{a}^{4}$), it is critically important to accurately determine vessel radius at the stenosis. The main limitation of improving radius estimation is the resolution of the X-ray angiography image. In an angiogram, the difference between a 70 and 75% stenosis cannot be distinguished since it is smaller than the pixel resolution, and thus the projections of both levels of stenosis appear identical. It is therefore difficult to differentiate between stenoses at a higher resolution even with a deep neural network. Super-resolution algorithms are a potential solution, as they have successfully been used to overcome the limitations of pixel size in magnetic resonance imaging^36–38^.

### Future work

Future work will focus on increasing the scope of the dataset to match real-world data more closely. In terms of synthetic data generation, we will expand our tree generator to incorporate further variations in the number and types of branches in the right and left coronary trees. One assumption of our synthetic dataset is that the patient is not moved between image acquisitions; however, in the catheterization lab, it is common practice to move the patient table during image acquisition to follow the flow of injected dye. Since the motion is controlled via a joystick and not often recorded, clinical angiograms are challenging to use as inputs for projection-based reconstruction methods because they require precise knowledge of image acquisition parameters. Neural networks may therefore provide a robust alternative for 3D reconstruction of coronary trees when those parameters are unknown. We can mimic the clinical workflow in our synthetic data generation by randomly translating the synthetic 3D coronary tree while generating binary angiography images. This would help our method to generalize better to clinical data since we would eliminate the assumption that the patient is not moved between image acquisitions. Radius reconstruction, particularly at the stenosis, can be improved by alternate strategies to encode the 3D coronary geometry and stenoses. Although we found that training a separate MLP to learn the radius along each branch led to better stenosis reconstruction, this strategy may become infeasible in tree structures with many branches. Changing the matrix representation of the coronary tree to emphasize stenoses could lead to better performance using one radius MLP. For example, the vessel radii could be encoded in a vector containing the proximal radius, distal radius, and parametric position and severity of the stenoses for each branch.

As discussed in "Reconstruction of coronary trees", we can improve centerline reconstruction by changing the loss function to incorporate more global characteristics of the vessels such curvature or tortuosity. Although we did not find that changing the backbone of our network led to significant improvements in centerline reconstruction in this work, it is possible that new developments in image feature extraction such as the focal modulation mechanism of FocalNet^39^ could benefit our multi-stage network as well.

Besides the synthetic projection images considered in this paper, our multi-stage neural network can be applied to clinical X-ray angiograms that have been processed with a segmentation algorithm to create binary input images. Clinical angiograms bring additional challenges, such as the motion of the coronary tree during the cardiac cycle. Care must be taken to identify angiographic frames acquired at the same point in the cardiac cycle to reconstruct a valid 3D coronary tree from clinical images.

In conclusion, in this work we have presented a proof-of-concept of a multi-stage neural network that can be used for 3D reconstruction of coronary trees from sets of uncalibrated X-ray angiography images, with sub-pixel resolution of vessel radius. We have demonstrated that reconstruction error is at an acceptable level to accurately model hemodynamic quantities such as pressure gradients across a vessel.

### Supplementary Information

Supplementary Information.

## Data Availability

The code for the synthetic coronary generator can be found on GitHub: https://github.com/kritiyer/vessel_tree_generator/.
