# Supplementary material for: A multi-stage neural network approach for coronary 3D reconstruction from uncalibrated X-ray angiography images
Source: Sci Rep. 2023 Oct 16;13:17603. doi: 10.1038/s41598-023-44633-2 (PMC10579444; doi:10.1038/s41598-023-44633-2)
Supplement: Supplementary file 1 — Supplementary Information. [file 41598_2023_44633_MOESM1_ESM.pdf]

Appendix A

Synthetic Coronary Tree Modeling Assumptions

Values derived from literature on coronary anatomy and clinical image data were used to inform the geometric modeling assumptions of the synthetic vessel tree generator. CTAs from 10 patients were additionally used to create a set of 3D vessel centerlines that formed the basis of spline-based synthetic centerline generation. All data were de-identified and collected retrospectively according to ethical guidelines, and the study protocol was approved by University of Michigan IRBMED (HUM00155491).

In each CTA image volume, 3D vessel centerlines were delineated for each branch of the coronary tree using the CRIMSON software package[1]. These centerlines were used to calculate vessel lengths and positions of each branch in the coronary tree. The relative position of a given branch with respect to the main right coronary was defined as the parametric position of its bifurcation point along the RCA.

We now list the anatomical assumptions that underpin our model:

1. The dimensions and relative position of each branch are informed by a combination of literature values [2] and the 10 CTA-derived patient geometries described above:

Table 1 – Parameters determining vessel dimensions and position

Branch	**Length [mm]**	**Maximum Diameter [mm]**	**Parametric Position**
RCA (+PLV)	120-140mm	4-5.5mm	N/A
SA	35-50mm	1.8-2.2mm	0.03-0.14
AM	48.5-78mm	2-2.4mm	0.22-0.4
PLV	40-60mm	1.9-2.5mm	0.59-0.72

2. Vessel radius
    a. We assume that the vessels have circular cross-sections (radially symmetric)
    b. The radius decreases linearly along the length of the vessels. The value of tapering for each branch is randomly sampled from a uniform distribution
        i. 60-70% along the main RCA, as the distal diameter is typically 1.5-2mm [2]
        ii. 30-50% in the side branches
3. Stenosis characteristics
    a. Following the work of Lee and Fung [3], we assume the stenosis has a gaussian profile
    b. Stenosis length ranges from 6-22mm [4]
    c. Local diameter reduction, or stenosis severity, ranges from 20-90% which aligns with clinical standards for mild, moderate, and severe stenoses [5]
    d. Although stenoses can present as concentric or eccentric lesions [6], we assume concentric stenoses in this model
    e. Stenoses are introduced in the RCA, PDA, and PLV branches, as stenoses in other branches are not typically treated with interventions
        i. 0-2 stenoses on the RCA+PLV and 0-1 stenoses on PDA
    f. We assume stenoses do not occur at bifurcation points

Appendix B

Hemodynamics Simulation Set Up

Computational fluid dynamics simulations were performed to compare pressure drop in ground truth and reconstructed coronary trees. In order to perform a simulation, we require the 3D geometry of the patient's blood vessel and information about pressure or flow to use as boundary conditions. The 3D geometry of each coronary tree was generated by importing the vessel centerlines and radius contours from our ground truth and predicted vessel matrices and lofting them to create solid 3D analytical models of each coronary tree. The 3D geometry was then discretized into an anisotropic finite element mesh composed of tetrahedral elements. The 3D Navier Stokes equations were solved on the nodes of this mesh to derive velocity and pressure. In these simulations, the density of the fluid ρ was set to 1.06e10^{-3} g/mm^3 and the viscosity was set to 0.004 g/(mm-s).

Since these coronary trees were synthetically generated, there were no clinical data to inform patient-specific boundary conditions; therefore, literature values [7] and allometric scaling laws [8], [9] were used instead. Identical boundary and initial conditions were applied to the ground truth and reconstructed coronary trees, namely:

1) 2x hyperemic steady flow at the inlet (0.2 L/min)
2) Resistances at the outlets, whose values were tuned using literature and allometric scaling laws
3) No slip at all walls
4) Initial mean arterial pressure of 80mmHg

Although coronary flow is pulsatile in nature, we can model the blood flow as steady since we are interested in the average pressure drop across the coronaries under hyperemic conditions, or conditions of maximum flow. Steady flow has previously been validated as sufficient to accurately estimate pressure drop across a stenosis [10]. Another assumption in our simulation is that the walls of the vessels are rigid and do not undergo large deformations. These simplifying assumptions greatly reduce computational cost. Simulations were run on 36 cores for 7-12 minutes.

References

[1] C. J. Arthurs *et al.*, "CRIMSON: An open-source software framework for cardiovascular integrated modelling and simulation," *PLOS Computational Biology*, vol. 17, no. 5, p. e1008881, May 2021, doi: 10.1371/journal.pcbi.1008881.

[2] B. F. Waller, C. M. Orr, J. D. Slack, C. A. Pinkerton, J. V. Tassel, and T. Peters, "Anatomy, histology, and pathology of coronary arteries: A review relevant to new interventional and imaging techniques—Part I," *Clinical Cardiology*, vol. 15, no. 6, pp. 451–457, 1992, doi: 10.1002/clc.4960150613.

[3] J.-S. Lee and Y.-C. Fung, "Flow in Locally Constricted Tubes at Low Reynolds Numbers," Mar. 1970, doi: 10.1115/1.3408496.

[4] L. Mauri *et al.*, "Effects of stent length and lesion length on coronary restenosis," *Am J Cardiol*, vol. 93, no. 11, pp. 1340–1346, A5, Jun. 2004, doi: 10.1016/j.amjcard.2004.02.027.

[5] J. A. Rumberger, "Coronary Artery Disease: A Continuum, Not a Threshold," *Mayo Clinic Proceedings*, vol. 92, no. 3, pp. 323–326, Mar. 2017, doi: 10.1016/j.mayocp.2017.01.009.

[6] B. F. Waller, “The eccentric coronary atherosclerotic plaque: Morphologic observations and clinical relevance,” *Clinical Cardiology*, vol. 12, no. 1, pp. 14–20, 1989, doi: 10.1002/clc.4960120103.
[7] S. Sakamoto *et al.*, “Relation of Distribution of Coronary Blood Flow Volume to Coronary Artery Dominance,” *The American Journal of Cardiology*, vol. 111, no. 10, pp. 1420–1424, May 2013, doi: 10.1016/J.AMJCARD.2013.01.290.
[8] G. B. West, J. H. Brown, and B. J. Enquist, “A General Model for the Origin of Allometric Scaling Laws in Biology,” *Science*, vol. 276, no. 5309, pp. 122–126, Apr. 1997, doi: 10.1126/science.276.5309.122.
[9] A. G. van der Giessen *et al.*, “The influence of boundary conditions on wall shear stress distribution in patients specific coronary trees,” *Journal of Biomechanics*, vol. 44, no. 6, pp. 1089–1095, Apr. 2011, doi: 10.1016/j.jbiomech.2011.01.036.
[10] R. E. Mates, R. L. Gupta, A. C. Bell, and F. J. Klocke, “Fluid dynamics of coronary artery stenosis.,” *Circulation Research*, vol. 42, no. 1, pp. 152–162, Jan. 1978, doi: 10.1161/01.RES.42.1.152.
